# Recirculation in Veno-Venous Extracorporeal Membrane Oxygenation

**DOI:** 10.3390/medicina60121936

**Published:** 2024-11-25

**Authors:** Veronica Gagliardi, Giuseppe Gagliardi

**Affiliations:** 1 Department of Anesthesiology and Intensive Care, University of Padua, 35129 Padova, Italy; giuseppe.gagliardi@aulss5.veneto.it; 2 Department of Anesthesiology and Intensive Care, Rovigo Hospital, aUlss5 Polesana, 45100 Rovigo, Italy

**Keywords:** veno-venous ECMO, recirculation, oxygen delivery

## Abstract

This review focuses on recirculation in the context of Veno-Venous Extracorporeal Life Support in adults. The methods employed to calculate and quantify the extent of recirculation, as well as factors affecting recirculation and interventions that could reduce recirculation, are detailed. As recirculation may significantly reduce extracorporeal oxygen delivery, leading to refractory hypoxemia, detecting and quantifying the recirculation fraction is fundamental in order to optimize VV-ECMO lung support. Although it is necessary to assess extracorporeal oxygen delivery, quantifying the amount of recirculation may be difficult. Besides mathematical methods, different experimental techniques for the direct measurement of recirculation are in development at present. Moreover, specific interventions and ECMO configurations could significantly reduce recirculation, and innovative systems are under study in this regard. Nevertheless, further human studies are needed to validate and standardize their use in clinical practice, and there remain limited data on their effectiveness and safety. More pre-clinical and clinical studies are required to assess the results obtained thus far and to improve the technologies to minimize the potential complications associated with their use.

## 1. Introduction

Veno-venous extracorporeal membrane oxygenation (ECMO) is the rescue therapy for severe acute respiratory failure, providing adequate oxygenation in the context of refractory hypoxemia. Moreover, while correcting hypoxemia, hypercapnia, and, consequently, acidosis, it allows for lung-protective ventilation [1].

In this extracorporeal support, venous blood is drained from the central venous compartment, oxygenated and decarboxylated by an extracorporeal membrane lung, and then reinfused into a central vein [2]. Therefore, vascular cannulation with either two cannulae (in a femoral–femoral or femoral–jugular configuration) or one single dual-lumen cannula (a device able to perform drainage and reinfusion) is required [3,4,5].

The assessment of the underlying disease is a fundamental process, as extracorporeal support is suitable for use in patients affected by potentially reversible diseases, representing a bridge to recovery. If the lung disease is chronic and irreversible, extracorporeal support should be employed only if patients are eligible for lung transplantation. The only absolute contraindication to VV-ECMO for respiratory failure is the presence of an irreversible underlying condition in a patient ineligible for lung transplantation. Moreover, relative contraindications consist of risk factors associated with overall poor prognosis despite the employment of VV-ECMO, such as contraindication to anticoagulation, irreversible neurologic injury, or untreatable metastatic cancer [6].

Besides the residual lung function, the main determinants of effective ECMO treatment—namely, one which achieves adequate arterial oxygen saturation and delivery—depends on both patient- and therapy-related factors. These elements include the extent of blood flow through the device, its ratio to the cardiac output of the patient, the oxygenator membrane performance (determined by the diffusion properties and the surface area), the fraction of oxygen in the sweep gas, the hemoglobin concentration, the metabolic demand and oxygen consumption, and the amount of recirculation within the ECMO circuit [2,7,8].

Furthermore, during ECMO support, ventilator pressures are usually reduced to avoid further mechanical ventilator-induced lung injury. This leads to different degrees of collapse of the lungs and, therefore, to almost total dependency on the ECMO circuit, where the left ventricular and arterial oxygen saturation are close to the pulmonary artery saturation [9]. In this context, the use of ECMO support can induce a significant drop in pulmonary artery pressure, weakening the adaptive pulmonary response of hypoxic vasoconstriction [10].

Both empirical experience and data from the literature indicate that providing adequate oxygenation requires an extracorporeal blood flow of roughly two-thirds of the native cardiac output. In severe cases of hypoxemic respiratory failure, arterial oxygen saturation may remain low despite the provision of extracorporeal support. Although the blood leaving the oxygenator in the ECMO circuit is fully saturated, blood reaching the tissues is mixed with venous blood, and its saturation depends on the aforementioned factors. In this context, a higher ECMO circuit blood flow also increases arterial oxygenation saturation, despite increasing recirculation [1].

**Figure 1:**
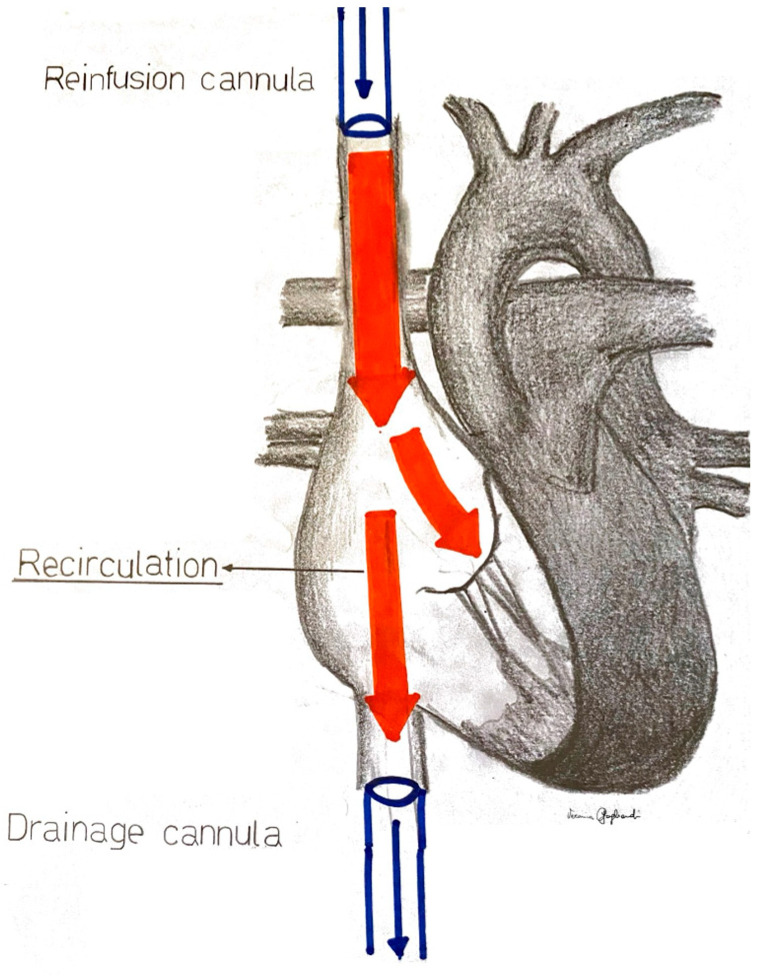
In recirculation, a certain amount of oxygenated blood coming from the extracorporeal circuit is directly withdrawn from the reinfusion cannula into the drainage cannula, therefore not reaching the systemic circulation through the tricuspid valve.

In particular, during VV-ECMO, a certain amount of oxygenated blood coming from the extracorporeal circuit is directly withdrawn from the reinfusion cannula into the drainage cannula, therefore not reaching the systemic circulation and the peripheral tissues. This dynamic phenomenon, called recirculation, is due to several factors, such as the positions of the cannulae on the patient and the hemodynamic interactions between the patient and the ECMO circuit. Recirculation may significantly reduce extracorporeal oxygen delivery, leading to refractory hypoxemia [11]. In ARDS, pulmonary artery hypertension (PAH) is common, which can lead to right ventricle failure. It has a multifactorial etiology, including pulmonary emboli and/or fibrosis [7]. In this framework, increased right-ventricular end-diastolic filling pressures (and the consequent tricuspid regurgitation) can redirect infused blood flow toward the drainage cannula, increasing the blood recirculation fraction (Figure 1).

In conclusion, detecting and quantifying the recirculation fraction is fundamental in order to optimize VV-ECMO lung support. Recirculation reduces the amount of oxygenated blood delivered to the patient, as a certain volume of blood does not take part in the gas exchange. Given the reduced concentration gradient of oxygen occurring in the ECMO membrane oxygenator, the effectiveness of the system is also reduced, causing a lower amount of oxygen to be delivered to the patient [1,2,12].

Although determination of the recirculation fraction is necessary to assess extracorporeal oxygen delivery, quantifying the amount of recirculation can be difficult [12,13].

The empirical range of acceptable recirculation is approximately 10–30%, although a standardized threshold is not defined. If it is higher than this range, reduced extracorporeal oxygen transfer occurs [8]. The degree of recirculation has been reported to vary from 2% to 57% [14]. In their study, Gehron et al. reported that the frequency recirculation data showed a median recirculation rate between 14 and 16% with a maximum rate of approximately 58%. Nevertheless, high recirculation rates above 35% were noted in only 13–14% of all measurements [8].

## 2. Data Collection

We performed a search of the literature consisting of all published manuscripts in PubMed. We selected articles using the phrase “Recirculation in Veno venous ECMO”. We obtained 54 results, from which we excluded articles dealing with the pediatric population and those that do not describe or study recirculation as the main topic.

We included the most relevant, complete, and recent articles dealing with recirculation in veno-venous ECMO, including both clinical and pre-clinical studies and review articles.

This review presents a synthesis of data and recommendations for the management of recirculation in adult patients disclosed in the existing literature.

## 3. Calculating the Amount of VV-ECMO Recirculation

Firstly, the recirculation fraction can be determined by comparing the extracorporeal membrane oxygenation (ECMO) drainage cannula oxygen saturation (SpreO2) to the patient’s venous oxygen saturation [2]. If there is no recirculation, these two values are close; on the other hand, when recirculation occurs, the oxygen saturation detected in the drainage cannula is increased in proportion to the recirculation [12].

Secondly, assessment of the trends of SpreO2 and peripheral arterial oxygen saturation (SaO2) could provide information about recirculation. An increase in SpreO2 in a context where SaO2 is decreased may indicate clinically significant recirculation. This is more relevant when SpreO2 exceeds SaO2—a situation occurring in cases of important recirculation.

If an intervention to reduce recirculation is effective, it leads to an increase in SaO2, associated with a decrease in SpreO2 [13,15].

However, to be more accurate in determining recirculation, we can calculate it using the following formula based on the oxygen content, expressed in terms of the oxygen saturation:

$$\mathrm{Recirculation}\;(\%)=\frac{\mathrm{SpreO}2-\mathrm{SvO}2}{\mathrm{SpostO}2-\mathrm{SvO}2}\times 100\%$$

where SpreO2 indicates the oxygen saturation in blood entering the draining cannula, SpostO2 denotes the oxygen saturation in blood leaving the infusing cannula, and SvO2 indicates the saturation of the venous blood just before entering the extracorporeal circuit and being oxygenated by ECMO.

SpreO2 and SpostO2 can be directly measured through blood gas analyses of blood entering and exiting the oxygenator, respectively.

If SpreO2 is equal to SvO2, the recirculation fraction is 0%. On the contrary, if SpreO2 is equal to SpostO2, the recirculation is 100%.

The main limitation of this approach in quantifying recirculation is the determination of SvO2. In fact, it does not correspond to the mixed venous saturation usually obtained by sampling blood from the Swan–Ganz catheter placed in the pulmonary artery. The blood sample from this site includes oxygenated blood reinfused by the ECMO circuit.

Hence, the proposed solutions to quantify SvO2 include the CVL method and the SvO2 method.

The CVL method consists of measuring the venous saturation of blood sampled from either the superior vena cava or the inferior vena cava using a central venous catheter. Although this value can easily be measured, it does not correspond to the mixed venous saturation and does not take into account the difference in oxygen content between the superior vena cava (SVC) and inferior vena cava (IVC) [15].

Indeed, the SvO2 method, as suggested by Van Heijst et al., [13] consists of temporarily ceasing the ECMO sweep gas flow, then using the ventilator to achieve an arterial oxygen saturation equivalent to the one achieved with ECMO support. The oxygen saturation of blood in the venous drainage cannula represents an accurate estimate of the true mixed venous oxygen saturation SvO2.

Nevertheless, although accurate, performing this measurement could be difficult and potentially dangerous, especially for patients who are highly dependent on extracorporeal support for gas exchange, who may not endure an interruption in the ECMO circulation [15,16].

### 3.1. The Ultrasound Dilution Method

The ultrasound dilution method is a potential alternative means which may provide a rapid, simple, reliable, and non-invasive method for assessing recirculation, even if it is potentially operator-dependent. This technique analyzes the ultrasound wave velocity properties and dilution of blood in the extracorporeal circuit to estimate the amount of recirculation through a non-invasive approach [13], detecting changes in the ultrasound velocity in response to saline injection. Ultrasonic flow sensors are placed on the drainage and injection cannulae.

After the injection of a saline bolus into the injection cannula, the velocity properties of ultrasound are altered, producing a “dilution curve”. Recirculated saline subsequently generates a second dilution curve, detected by the ultrasonic probe on the drainage cannula [16].

The ratio of these two velocities, represented as the areas under the two dilution curves (AUC US, ultrasound), allows for calculation of the recirculation in the circuit:

$$R=\frac{\mathrm{AUCinfusion}\;\mathrm{cannulaUS}}{\mathrm{AUCdrainage}\;\mathrm{cannulaUS}}\times 100\%$$

If the dilution curve is not detected in the drainage cannula, it means that the recirculation is null; meanwhile, if the same dilution curve is detected in both the drainage and the reinfusion cannulae, the recirculation is 100%.

Pre-clinical studies have compared ultrasound dilution with both the CVL and SvO2 techniques at progressively increasing blood flow rates in an animal model. The ultrasound dilution recirculation rates were more similar to those detected with the SvO2 method than the CVL method [15]. The ELSA^®^ monitor has been developed, but it is not in widespread use in clinical practice and more studies are needed to validate the associated results [17]. The ELSA monitor provides a non-invasive method to measure recirculation in VV ECMO without blood sampling. ELSA Flow/Dilution Sensors use ultrasonic transit-time sensors to measure the delivered volume flow in fluids, positioned in the drainage and reinfusion cannulae. When a bolus of saline is injected into the circuit in the reinfusion cannula, the Transonic^®^ ELSA Monitor detects and quantifies recirculation. This device enables a potentially standardized method to make ultrasound a reproducible technique.

### 3.2. Thermodilution

Thermodilution to determine cardiac output is unreliable in the context of veno-venous extracorporeal membrane oxygenation, as part of the cold indicator is drawn into the extracorporeal circuit. In this framework, thermodilution might be employed to measure recirculation by injecting a bolus into the reinfusion cannula and measuring the temperature–time curve occurring in the drainage cannula, representing a potential non-invasive way of quantifying recirculation [16].

Therefore, in pre-clinical ex vivo settings, modified thermodilution techniques have been studied to assess both the native cardiac output and recirculation fraction. These studies demonstrated that an adapted thermodilution technique may enable the estimation of recirculation, and, if the recirculation fraction is less than or equal to 40%, a clinically acceptable cardiac output value could be detected [2]. These studies were performed by positioning two thermocouples along the circuit: one on the ECMO reinfusion cannula and the other on the drainage cannula. The recirculation fraction was calculated as the ratio between the areas under the curves (AUC T, temperature) of the temperature traces:

$$R=\frac{\mathrm{AUCinfusion}\;\mathrm{cannulaT}}{\mathrm{AUCdrainage}\;\mathrm{cannulaT}}\times 100\%$$

Further in vivo studies are necessary to confirm these results [11,14].

### 3.3. Lithium Dilution

Lithium dilution was tested and validated ex vivo. The lithium concentration is measured both in the ECMO drainage and reinfusion cannulae after the injection of a known quantity of lithium chloride into the reinfusion cannula. The recirculation fraction is estimated as the ratio between lithium concentrations in the ECMO cannulae. Lithium dilution techniques may have important limitations due to the accumulation of lithium within the bloodstream [18].

## 4. Factors Affecting Recirculation

Recirculation is influenced by several factors, such as the cannulation configuration, positioning and size of cannulae, direction of the reinfused blood flow, ECMO blood flow, pump speed, intrathoracic and intra-abdominal pressures, patient positioning (e.g., rotation of the head and neck), volume status, and cardiac output [16].

### 4.1. Cannulation Configuration, Positioning, and Cannula Size

Considering the femoral-to-internal jugular VV-ECMO configuration, a certain amount of recirculation is unavoidable, as the reinfusion jet is directed toward the drainage port. The proximity and the direction of the reinfusion and drainage cannulae have a direct impact on the amount of recirculation, with a higher recirculation fraction when the two ports are closer, whereas a broader position can decrease recirculation. Relatively larger drainage cannulae may keep the same blood flow rates at a lower pump speed, with less negative venous pressure in the drainage cannula, potentially reducing the extent of recirculation.

### 4.2. Pump Speed, Extracorporeal Blood Flow, and Cardiac Output

There is a direct correlation between pump speed and recirculation: the higher the pump speed, the higher the amount of recirculation.

Moreover, recirculation may be affected by the cardiac output (CO) of the patient and its ratio to the ECMO blood flow. A cardiac output close to the extracorporeal blood flow increases recirculation, while a cardiac output higher than the extracorporeal blood flow decreases recirculation. Nevertheless, both conditions can lead to systemic hypoxia, as they reduce the fraction of venous blood oxygenated by the extracorporeal system. To avoid this reduction in oxygen delivery, it has been recommended to use a ECMO blood flow of approximately two-thirds of the CO in clinical practice [8].

This highlights how recirculation is a dynamic process, and considering the ratio between the CO and the ECMO blood flow is necessary. Additionally, although low recirculation due to high CO may increase extracorporeal oxygen delivery (DO2), the mismatch between higher CO and lower pump flow can lead to pre-pulmonary shunting and refractory patient hypoxemia [8,19,20]. Therefore, recirculation must be assessed repeatedly in order to optimize these parameters.

### 4.3. Intrathoracic, Intracardiac, and Intra-Abdominal Pressures

Pressures in the different districts have different effects on the extent of recirculation. Increases in thoracic volumes and decreases in chest wall compliance lead to increases in central venous, pericardial, and pleural pressures, thus reducing right and left cardiac output.

If significantly elevated intrathoracic pressures occur, venous return to the heart may be impaired. Therefore, the reinfused blood flow is preferentially directed toward the drainage cannula, increasing the recirculation fraction. In extreme circumstances, ECMO blood flow may cease as a consequence of the severe reduction in venous return.

On the contrary, high intra-abdominal pressures may cause significant compression of the IVC, potentially limiting drainage and, in extreme circumstances, ceasing the ECMO support. These effects may decrease the extent of recirculation, although the decreased flow into the circuit would lessen the systemic oxygenation [8,21,22] (see Table 1).

## 5. Interventions to Reduce Recirculation

### 5.1. Modifying the Positions of the Cannulae

Considering the femoral–jugular configuration, a standard distance that should be maintained between the cannulae ports has not been defined. If clinically relevant recirculation is detected, one solution is to increase the distance between the drainage and reinfusion cannulae by withdrawal of one of the two cannulae. In this context, data from the literature recommend that the tip of the femoral drainage cannula should be positioned near the atrial–IVC junction with the side ports located within the hepatic IVC. This portion is least collapsible—due to the surrounding hepatic parenchyma—and, therefore, least likely to impair drainage through occlusion of the ports when negative pressure due to the pump speed occurs. This could limit the withdrawal of the drainage cannula.

The return cannula, instead, should be positioned at the junction between the superior vena cava and the right atrium. While manipulating the cannulae, the blood flow should be checked carefully.

The drainage cannula and the reinfusion cannula should be placed apart by at least 10–13 cm to minimize recirculation, as recommended in the published literature.

In cases of bi-femoral cannulation, placing the tip of the drainage cannula in the inferior vena cava and the tip of the return cannula in the right atrium is recommended [23].

However, data from the literature suggest that positioning the drainage cannula in the SVC could be possible without cannula modification and additional cannulation; this alternative position of the cannulae in VV-ECMO can provide sufficient full support even in a patient with a low intravascular volume or high intrathoracic/intra-abdominal pressure. The highlight of this positioning technique is that the lowest recirculation rate can be identified using dilution ultrasound monitoring [24].

### 5.2. Inserting an Additional Drainage Cannula

The addition of a second drainage cannula may achieve comparable blood flow rates at lower pump speeds, resulting in the generation of less negative venous pressure.

Studies have demonstrated that the utilization of VV-V ECMO with bicaval drainage (i.e., with the addition of a second upper-extremity drainage cannula, inserted from the left subclavian vein) could be safe and effective in improving oxygen saturation in patients undergoing VV-ECMO suffering from refractory hypoxemia. Oxygenation is improved due to higher flows occurring with similar pump speed, without an increase in recirculation [25]. Complications could include damage to the anatomical structure and an increased risk of cannula-related infection, which could be minimized if the sealed dressing of the cannula sites is maintained with a sterile procedure.

### 5.3. Manipulation of the Shape of the Reinfusion Cannula

It has been demonstrated that modifying the shape and orientation of the reinfusion cannula may decrease the extent of recirculation. First, in one study, sutures were employed to create curvature in the cannula to direct the reinfusion jet toward the tricuspid valve. A reduction in recirculation occurred and, contextually, an improvement in arterial oxygenation was observed.

A configuration proposed in another study involved the combination of a curved reinfusion cannula directed toward the tricuspid valve and placement of the venous drainage cannula with its distal tip in the SVC–right atrial junction. This cannulation strategy, named the x-configuration, was studied in a small, randomized controlled trial and was associated with improved systemic oxygenation. Nevertheless, the drainage cannula employed in the study had multiple side holes. Differences in the shape, number, spacing, and size of the side holes of the drainage cannula may have contributed to improving the performance of the extracorporeal oxygenator [26].

However, this configuration also has certain limitations: modifying the return cannula might lead to tricuspid valve injury or tricuspid regurgitation if the cannula is positioned through the tricuspid valve.

### 5.4. Use of the Bicaval Dual-Lumen Cannula (DLC)

The bicaval dual-lumen cannula is one of the most significant advancements in cannula design; it is effective in reducing recirculation and is considered the most effective method in this regard.

The original design of the cannula dates back to the 1980s: it was designed to be unicaval, with its tip terminating in the right atrium, and the drainage and reinfusion ports were in relatively close proximity. Since then, it has undergone several modifications, and the new design (Wang–Zwische dual-lumen cannula) consists of a bicaval design (the distal tip is located in IVC) with drainage ports located in both the SVC and the IVC, and with the reinfusion jet directed toward the tricuspid valve. Hence, it significantly reduces the amount of recirculation, which has been reported to be as low as 2% in an acute setting [15,27], even though it increased to as high as 40% over days of VV-ECMO therapy [27]. However, besides the advantage of using a single vascular access, it also has several disadvantages.

One such disadvantage is the high risk of malposition of the cannula, which can significantly increase the amount of recirculation and compromise the effectiveness of the assistance, with recirculation rates reported to be around 50% in an animal model. Any adjustment of the cannula after its initial insertion should, therefore, be performed under echocardiographic guidance to ensure the appropriate orientation of the reinfusion jet [26]. This device is also associated with an increased risk of cardio-vascular perforation [12,28,29]. Another limitation of this device is the risk of bleeding and thrombosis, as the side-holes of the cannula lead to significant non-physiological wall shear stress, which cause platelet activation and impaired hemostasis. A study identified that the IVC and SVC backflows at late systole and late diastole cause the maximum wall shear stress in correspondence to the side holes of the cannula. As such, the best-performing cannula was a DLC with 3 and 16 side holes within the SVC and IVC, respectively, which was able to significantly reduce the average wall shear stress without causing recirculation [30].

### 5.5. Performing Veno-Right-Ventricular Cannulation

Another modification of the traditional femoral–jugular configuration consists of maintaining the drainage from the IVC and positioning the reinfusion cannula in the right ventricle through the tricuspid valve, performed percutaneously through the jugular vein. The veno-right-ventricular ECMO showed significantly less recirculation in a study dealing with a canine model. Nevertheless, positioning the reinfusion cannula into the right ventricle has been found to be associated with endocardial damage and arrhythmias. Therefore, specialized catheters should be developed in order to reduce such injuries [31,32,33] (see Table 2).

## 6. Conclusions

Recirculation is a common, multifactorial, and dynamic event occurring during veno-venous extracorporeal therapy. As it can lead to severe refractory hypoxemia, its recognition and management are fundamental concerns.

At present, the precise amount of recirculation is difficult to quantify, and its measurement is mainly performed using mathematical approximations.

Although methods for the systematic and direct clinical measurement of recirculation are increasingly studied, further human studies are needed to validate and standardize their use in clinical practice.

Although alternative cannulation strategies and devices have been suggested to minimize recirculation, there are still limited data on their effectiveness and safety.

## 7. Further Directions

More pre-clinical and clinical studies are required to assess the results obtained thus far and to improve the technologies to minimize the potential complications. Further research should be performed on the ELSA monitor, a non-invasive device that could provide a reproducible means to quantify recirculation. Furthermore, studying the correct positioning of the dual-lumen cannula, as well as defining a standardized procedure employing different devices such as echocardiography and/or fluoroscopy, could reduce the complications related to such devices.

## Figures and Tables

**Table 1:** Factors affecting recirculation.

Cannula size	Relatively larger drainage cannulae keep the same blood flow rates at a lower pump speed, with less negative venous pressure in the drainage cannula, potentially reducing the extent of recirculation.
Cannula configuration and position	A higher recirculation fraction occurs when the two ports are closer, whereas a broader position can decrease recirculation
Pump speed	The higher the pump speed, the higher the amount of recirculation
Extracorporeal blood flow and cardiac output	A cardiac output close to the extracorporeal blood flow increases recirculation, while a cardiac output higher than the extracorporeal blood flow decreases recirculation
Intrathoracic pressure	If significantly elevated intrathoracic pressures occur, venous return to the heart may be impaired. Therefore, the reinfused blood flow is preferentially directed toward the drainage cannula, increasing the recirculation fraction.

**Table 2:** Interventions to reduce recirculation.

Modifying the positions of the cannulae	One solution is to increase the distance between the drainage and reinfusion cannulae by withdrawal of one of the two cannulae
Inserting an additional drainage cannula	The addition of a second drainage cannula may achieve comparable blood flow rates at lower pump speeds, resulting in the generation of less negative venous pressure.
Manipulation of the shape of the reinfusion cannula	It has been demonstrated that modifying the shape and orientation of the reinfusion cannula may decrease the extent of recirculation.
Use of the bicaval dual-lumen cannula (DLC)	The bicaval dual-lumen cannula is one of the most significant advancements in cannula design; it is effective in reducing recirculation and is considered the most effective method in this regard.
Veno-right-ventricular cannulation	Another modification of the traditional femoral–jugular configuration consists of maintaining the drainage from the IVC and positioning the reinfusion cannula in the right ventricle through the tricuspid valve, performed percutaneously through the jugular vein.
